# Amphiphilic aminoglycosides: Modifications that revive old natural product antibiotics

**DOI:** 10.3389/fmicb.2022.1000199

**Published:** 2022-09-23

**Authors:** Jon Y. Takemoto, Guillermo A. Altenberg, Naveena Poudyal, Yagya P. Subedi, Cheng-Wei T. Chang

**Affiliations:** ^1^Department of Biology, Utah State University, Logan, UT, United States; ^2^Department of Cell Physiology and Molecular Biophysics, Center for Membrane Protein Research, Texas Tech University Health Sciences Center, Lubbock, TX, United States; ^3^Department of Chemistry and Biochemistry, Utah State University, Logan, UT, United States

**Keywords:** amphiphilic aminoglycosides, kanamycin, neomycin, neamine, antibiotic resistance, connexin

## Abstract

Widely-used *Streptomyces*-derived antibacterial aminoglycosides have encountered challenges because of antibiotic resistance and toxicity. Today, they are largely relegated to medicinal topical applications. However, chemical modification to amphiphilic aminoglycosides can revive their efficacy against bacterial pathogens and expand their targets to other pathogenic microbes and disorders associated with hyperactive connexin hemichannels. For example, amphiphilic versions of neomycin and neamine are not subject to resistance and have expanded antibacterial spectra, and amphiphilic kanamycins are effective antifungals and have promising therapeutic uses as connexin hemichannel inhibitors. With further research and discoveries aimed at improved formulations and delivery, amphiphilic aminoglycosides may achieve new horizons in pharmacopeia and agriculture for *Streptomyces* aminoglycosides beyond just serving as topical antibacterials.

## Introduction

**FIGURE 1:**
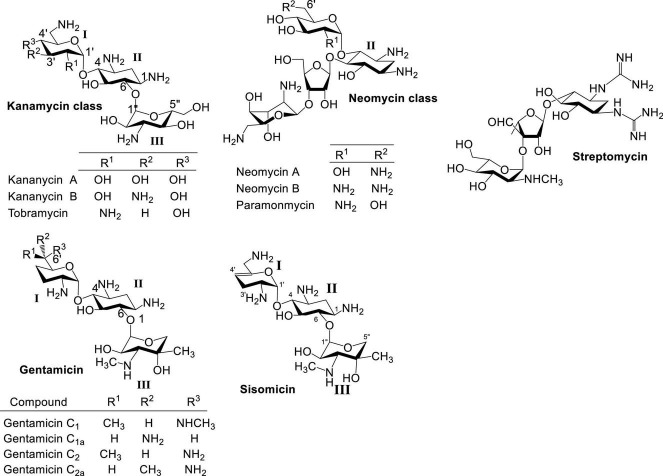
Structures of natural AGs.

Naturally occurring aminoglycosides (AGs) isolated primarily from *Streptomyces* sp. are important broad spectrum antibacterial agents that have been used clinically for decades (Figure 1; Umezawa and Hooper, 1982; Gonzalez et al., 1998; Arya, 2007; Krause et al., 2016). Besides streptomycin, neomycin and kanamycin have been two of the most studied and employed classes of AG antibiotics. Neomycin belongs to a group of AGs containing a 4,5-disubstituted 2-deoxystreptamine core (ring II), while kanamycin contains a 4,6-disubstituted 2-deoxystreptamine core. Other AG groups include gentamicin (Weinstein et al., 1963) and sisomicin (Weinstein et al., 1970), which structurally resemble kanamycin antibiotics but are produced by *Micromonospora* sp. Structurally, gentamicin and sisomicin usually contain deoxygenation at ring I, and have a different ring III aminosugar from kanamycin. The emergence of antibiotic resistant bacteria has significantly hampered the use of naturally occurring AGs.

**FIGURE 2:**
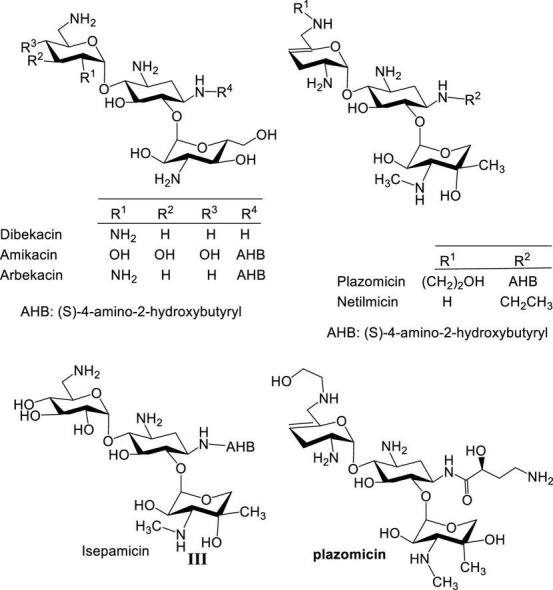
Structures of semisynthetic aminoglycosides.

During the 1970s, several semisynthetic AGs with improved activity against resistant bacteria were developed, such as amikacin (Kawaguchi et al., 1972), netilmicin (Campoli-Richards et al., 1989), isepamicin (Miller et al., 1978; Nagabhushan et al., 1978), arbekacin (Kobayashi et al., 1995), and most recently plazomicin (Aggen et al., 2010; Figure 2). However, these new AGs were limited when confronted with bacterial resistance arising from overexpression of AG-modifying enzymes. Over a hundred different AG-modifying enzymes are known to inactivate AGs by introducing structural modifications at various AG sites (Ramirez and Tolmasky, 2010; Garneau-Tsodikova and Labby, 2016). Some display substrate promiscuity and modify both neomycin and kanamycin classes (Fong and Berghuis, 2002). In addition, ototoxicity and nephrotoxicity often associated with AGs persist despite efforts devoted to lowering these side effects (Xie et al., 2011; Kovacik et al., 2021). Finally, as a result of the prevalence of AG-modifying enzymes, more structural motifs are needed at specific positions of AGs which adds to the challenges and cost of synthesizing new AGs. As an example, an expensive AG, sisomicin, was used as starting material for the development of plazomicin to circumvent part of the problem of regioselective chemical modification, which even at a relatively concise synthesis resulted in a cost >$3,000 US dollars per 100 mg.

An alternative approach is to revive and repurpose AGs using cost-effective AGs like neomycin or kanamycin as feedstocks for the synthesis of amphiphilic AGs (AAGs) (Chang et al., 2010; Chang and Takemoto, 2014; Domalaon et al., 2018; Subedi et al., 2018; Dezanet et al., 2020). AGs are hydrophilic, and the attachment of hydrophobic groups creates AAGs. In general, AAGs have two distinct features that make them different from AGs: broader antimicrobial profile and altered mode of action. AGs are active against aerobic Gram-positive and Gram-negative bacteria but inactive against anaerobic bacteria and fungi. In contrast, AAGs have antibacterial—aerobic and facultative anaerobic—and antifungal activities. Traditional AGs display antibacterial activity by binding to 16S rRNA thereby interfering with protein synthesis, whereas AAGs exert bioactivity by primarily targeting microbial membranes (Udumula et al., 2013). The latter bioactivity allows AAGs to be active against bacteria that are resistant to AGs, and also against fungi.

In contrast to the semisynthesis of AGs, where regioselective incorporation of structural motifs is essential, the synthesis of AAGs can be performed at the sites that are most amenable to modification. This drastically alleviates the burden of chemical modification and the cost of production. The most cost-effective feedstocks of AGs are neomycin B and kanamycin A, and this review focuses on the applications and biological activities of AAGs derived from neomycin B, kanamycin A and neamine, a derivative from neomycin B. Particular attention is directed toward synthetic approaches as they dictate the costs of production and the feasibility of offering marketable products.

## Amphiphilic neamines and neomycins

**FIGURE 3:**
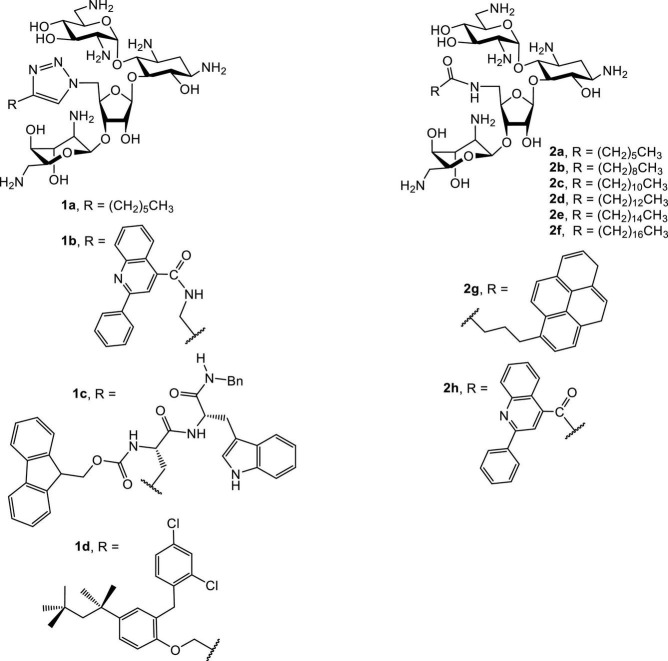
Structures of representative amphiphilic neomycins.

Several groups have reported the synthesis and antibacterial investigations of amphiphilic neomycins (Figure 3; Bera et al., 2008, 2010a; Zhang et al., 2008, 2009). The lead amphiphilic neomycins show broad spectrum and unusual biological activities, especially against bacteria resistant to AGs. For example, 5″-aminoneomycin with attached linear acyl groups displays prominent antibacterial activities against a panel of bacteria, including methicillin-resistant *Staphylococcus aureus* (MRSA) and vancomycin-resistant enterococci (VRE). The activity against VRE is of particular interest since facultative enterococci are intrinsically resistant against traditional AGs such as neomycin, amikacin, gentamicin, tobramycin and kanamycin due to the lack of AG-uptake mechanisms. Therefore, the unusual activities of AAGs, especially against VRE, strongly imply that the attachment of hydrophobic groups results in alteration of the traditional antibacterial mode of action.

The antibacterial minimum inhibitory concentrations (MICs) of amphiphilic neomycins are summarized in Tables 1, 2. In general, the amphiphilic neomycin derivatives in Table 1 are less active against Gram-negative bacteria. There is no clear structure-activity relationship (SAR) for the derivatives with aromatic motifs attached (**1b,c, 2g,h**), but the activity of **1c** against MRSA is of particular interest (Table 1). For the derivatives with linear alkyl chains attached (**1a, 2a-f**), increasing the hydrophobicity (lipophilicity) appears to enhance antibacterial activity, as **2e** (C16, hexadecyl) is the most active. Nevertheless, extending the alkyl chain further to C18 (octadecyl) decreases the activity slightly. The most significant findings are the unusual activities of **2e** against MRSA (Tables 1, 2) and VRE (Table 2). Traditional AGs are inactive or much less active against these two pathogenic bacterial strains. Amphiphilic neomycins with hydrophobic groups attached at the N-6‴ position have also been investigated (Mével et al., 2012), and trends of biological activities similar to those of other amphiphilic neomycins were observed.

**TABLE 1:** MICs (μg/mL) of 5″-modified neomycin AAGs against MRSA (Bera et al., 2008, 2010a).

Compound	*S. aureus* ^a^	MRSA^b^	*E. coli* ^c^	*E. coli* ^d^	*P. aeruginosa* ^e^
Neomycin B	1	256	4	8	512
**1c**	8	4	16	32	128
**1d**	8	8	16	64	128
**2c**	32	>512	64	64	256
**2e**	4	8	32	64	128
**2f**	16	32	128	64	>512
**2g**	16	256	32	64	256

^a^ATCC29213.

^b^ATCC33592.

^c^ATCC25922.

^d^ATCC6174 (gentamicin resistant).

^e^ATCC27853.

**TABLE 2:** MICs (μg/mL) of 5″-modified neomycin AAGs against MRSA and VRE (Zhang et al., 2008, 2009).

Compound	*S. aureus* ^a^	MRSA^b^	*E. coli* ^c^	*Enterococcus faecali* ^d^	*P. aeruginosa* ^e^
Neomycin B	1	125	4	≥250	64
Amikacin	1	8–16	1	≥250	0.5–1
Vancomycin	0.5–1	1	125–250	250	≥250
**1a**	4	ND^f^	32	ND	ND
**1b**	8	ND	32	ND	ND
**2a**	2–4	125	16	≥250	≥250
**2b**	16	125–250	32	64–125	16–32
**2c**	8–16	16–32	16–32	64–125	16
**2d**	2–4	4–8	8–16	8–16	8
**2e**	1–2	2–4	4–8	4	4
**2f**	2–4	2–4	4–8	8–16	8–16
**2h**	4–8	ND	8–16	ND	ND

^a^ATCC25923.

^b^ATCC33591.

^c^ATCC25922.

^d^ATCC51299 (vancomycin-resistant enterococci).

^e^ATCC27853.

^f^ND: not determined.

**FIGURE 4:**
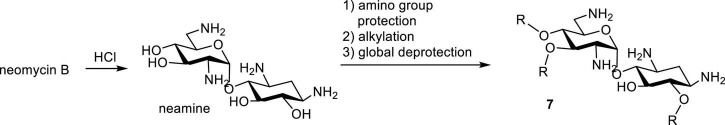
Synthesis of amphiphilic neamine.

Neamine is obtained by acid-hydrolysis of neomycin. Modification to amphiphilic neamine usually begins with the protection or masking of amino groups, for example tritylation followed with alkylation of hydroxyl groups and global deprotection (Figure 4; Benhamou et al., 2015; Mingeot-Leclercq and Décout, 2016; Zimmermann et al., 2016). In general, amphiphilic neamines have been noted for their unique activities especially against *Pseudomonas* sp (Mingeot-Leclercq and Décout, 2016; Zimmermann et al., 2016). This is significant as *Pseudomonas* sp. are known for their unusual membrane compositions and efflux resistance that compromise the activity of many antibiotics. Additionally, amphiphilic neamines with di-*N*-methylation have improved activities against Gram-negative bacteria (Benhamou et al., 2015).

Amphiphilic paromomycins have also been synthesized and employed as inhibitors against AG-modifying enzymes (Szychowski et al., 2011) and antibacterials toward Gram-positive pathogens (Berkov-Zrihen et al., 2013a). AAGs derived from streptomycin, gentamicin and sisomicin have not been reported, probably due to cost considerations and the challenges of site-selective modifications.

Since AAGs have been shown to have mode of antibacterial action different from tradidional AGs, several groups have engaged in investigations on their mode of action. Using atomic force microscopy, Mingeot-Leclercq and co-workers have reported that amphiphilic neamine derivatives bind to the lipopolysaccharides of *P. aeruginosa* and induce membrane depolarization (Mingeot-Leclercq and Décout, 2016). More evidence supporting this mode of action comes from studies using the fluorogenic dye SYTOX (Udumula et al., 2013). SYTOX cannot penetrate the intact bacterial membrane, but enters cells through pores formed in the bacterial membrane and binds nucleic acids which results in strong fluorescence. Following exposure to **6e**, *Escherichia coli* and *S. aureus* strains showed high levels of green fluorescence, whereas *S. aureus* cells treated with neomycin and SYTOX did not, as pore formation with traditional AGs is not expected.

It is interesting to note the similarities between the antibacterial activities of AAGs and lipopeptides, another class of amphiphilic compounds. Addition of acyl chains to peptides alters membrane lipid composition and packing. The antimicrobial activity of lipopeptides is associated with membrane modifications, including changes in its curvature, surface charge and dipole potential, as well response to changes in ionic strength (Balleza et al., 2019; Shahane et al., 2019; Juhi et al., 2021). Although details of the mode of action of AAG are still being deciphered, it is likely that they will resemble those of antimicrobial lipopeptides.

Among the reported investigations of amphiphilic neomycin and neamine, several studies found these compounds less toxic based on hemolysis experiments (Zhang et al., 2009; Benhamou et al., 2015). Compounds **2a**, **2e** and **2f** were found to be weakly antifungal with MICs ranging from 62.5 to 125 μg/mL against the wheat and barley pathogen *Fusarium graminearum* (Takemoto and Chang, 2010). However, little is known regarding the bioactivity of these compounds beyond their antibacterial activity.

## Amphiphilic kanamycins

**FIGURE 5:**
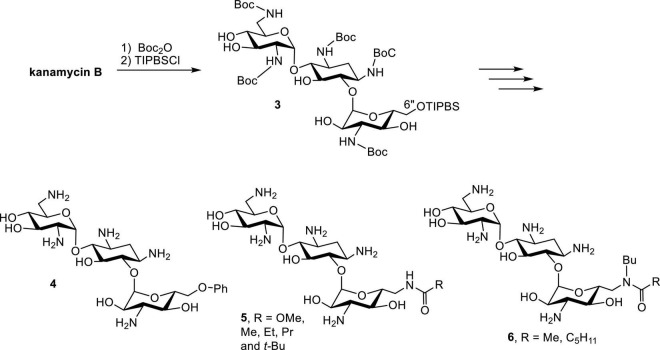
Synthesis of AKs.

Early successes in the synthesis of amphiphilic kanamycins (AK) employed kanamycin B as the starting material (Schepdael et al., 1991). Following the protection of Boc groups, the 6″-OH (the only primary hydroxyl group) can be selectively modified using a nucleophilic substitution approach (Figure 5). Derivatives with various functional groups, such as thioether, alkoxyl, alkylamino, alkylamido and esters, have been synthesized and evaluated for their antibacterial activities. Although these compounds manifest good antibacterial activity against AG susceptible strains, they do not display significant antibacterial activity against AG-resistant bacteria such as *Streptococcus faecalis* and *P. aeruginosa* (Schepdael et al., 1991).

**FIGURE 6:**
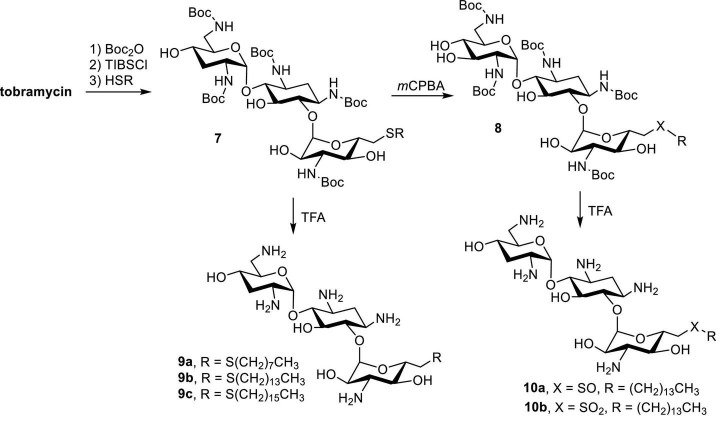
Synthesis of amphiphilic tobramycin.

Tobramycin, a kanamycin class AG, has been converted to 6″-modified amphiphilic derivatives using similar methods (Figure 6; Herzog et al., 2012). From the selected MICs, a long linear alkyl chain (C14 and C16) seems to improve antibacterial activity (Table 3). The most active compounds,8**a** and **8b**, inhibit growth of VRE and tobramycin-resistant *E. coli.* On the other hand, oxidation of alkylthio to sulfones or sulfoxides slightly decreases antibacterial activities.

**TABLE 3:** MICs of 6″-modified amphiphilic tobramycin derivatives (in μg/mL) (Herzog et al., 2012).

Compound	*S. epidermidis* ^a^	MRSA	VRE^b^	*E. faecalis* ^c^	*E. coli* ^d^
Tobramycin	0.3	>150	>150	150	>150
**8a**	75	>150	>150	>150	>150
**8b**	1.2	9.4	18.8	75	4.7
**8c**	4.7	9.4	18.8	18.8	4.7
**10a**	2.3	18.8	75	9.4	>150
**10b**	2.3	18.8	37.5	4.7	37.5

^a^ATCC12228.

^b^Vancomycin resistant enterococcus.

^c^ATCC29212.

^d^Tobramycin resistant (AAC(6′)/APH(2″)).

Other AK designs have been reported, including kanamycin A with a 6″-alkyltriazole group (Bera et al., 2010b), 4′,6″-dialkylthio tobramycin (Berkov-Zrihen et al., 2013b) and 6′-chelostolcarbonyl kanamycin A (Sainlos et al., 2003). In general, these compounds manifest broad spectrum antibacterial activities at low micromolar concentrations.

**FIGURE 7:**
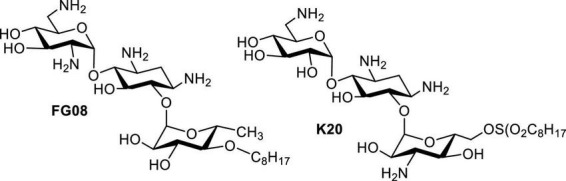
Structures of **FG08** and **K20**.

In 2006, it was reported that AGs such as neomycin, paromomycin, ribostamycin and streptomycin possess antifungal activities against *Phytophthora* and *Pythium* species (Lee et al., 2005). Inspired by this article, screening of libraries of neomycin, kanamycin and pyranmycin derivatives led to the identification of AK **FG08** as the most potent antifungal AG tested (Figure 7; Chang et al., 2010). Interestingly, **FG08** was inactive against bacteria, a discovery pointing to a new and significant bioactivity of AAGs and AKs with potential for broader applications in medicine and agriculture. Furthermore, the antifungal activity of **FG08** involved formation of pores in the plasma membrane (Shrestha et al., 2013). Therefore, attachment of hydrophobic groups to make AAGs not only expanded the antibacterial target species spectrum, but also uncovered a “switch” that adds antifungal activity to the AG antimicrobial profile.

Although **FG08** showed promising fungicidal activity against crop disease caused by Fusarium head blight (FHB), its complex synthesis makes it unsuitable for scale-up production, and therefore, practical applications for **FG08** in medicine and agriculture are limited. Based on the SAR investigation of **FG08** and related compounds (Fosso M. et al., 2015), a simplified protocol for the production of a 2nd generation antifungal AK (**K20**) was explored (Shrestha et al., 2014). In addition to showing broad spectrum antifungal activity, **K20** can be synthesized in 200–300 g scale batch (Table 4). Furthermore, **K20** also exerts strong synergism with commercially available crop fungicides (Shrestha et al., 2014, 2015b) and these **K20** combinations demonstrated efficacy in wheat field trials against FHB (Takemoto et al., 2018). In short, a combination of **K20** and crop fungicides can reduce FHB and production of the toxin deoxynivalenol that is associated with FHB. These findings suggest the possible use of **K20** in combination with established crop fungicides at lower concentrations, thus reducing costs, toxicities and environmental impacts of crop protection strategies.

**TABLE 4:** MICs (μg/mL) of **K20** and kanamycin A against bacteria and fungi^a^.

Strain	K20	Kanamycin A	ITC^b^	FLC^b^
*Cryprococcus neoformans* H99	3.9–7.8	>125	1.56	1.56
*C. neoformans* 94-2586	3.9–7.8	>125	0.06	1.56
*C. neoformans* 90-26	3.9–7.8	>250	0.37	>0.195
*C. pseudotropicalis* YOGI	15.6	>250	0.125–0.8	ND^c^
*C. lusitaniae* 95-767	>7.8	>250	0.2	1.56
*Candida rugosa* 95-967	15.6	>250	0.12	>0.78
*C. tropicalis* 95-41	15.6	>250	>25	>25
*C. albicans* 10231	15.6	>250	0.75	25
*C. albicans* 64124 (R)^a^	31.3	>500	>64	>200
*C. albicans* MYA 2876 (S)^a^	15.6	>250	>2	1.56
*C. albicans* B-311	>7.8	>250	16–32	>25
*C. albicans* 94-2181	>7.8	>250	>8–16	>12.5
*C. parapsilosis* (R)	15.6–31.3	>250	0.5	>16
*C. parapsilosis* (S)	15.6	>250	0.015	0.12
*Fusarium graminearum* B-4-5A	7.8	>125	ND	ND
*F. oxysporum*	31.3	>250	ND	ND
*Aspergillus flavus*	300	>250	0.125	ND
*A. niger*	>150	>250	ND	ND
*B. alcada*	15.6	ND	ND	ND

^a^(R) Resistant, (S) Sensitive.

^b^FLC, fluconazole; ITC, itraconazole.

^c^ND, not determined.

**FIGURE 8:**
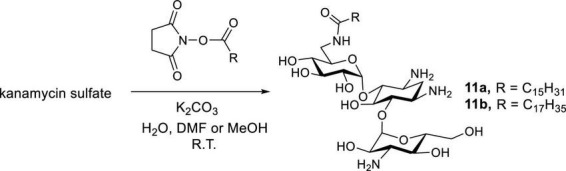
One-step synthesis of AKs **11a** and **11b**.

Newer and improved antifungal AK designs have been reported since the discovery of **K20** (Fosso M. Y. et al., 2015; Shrestha et al., 2015b; Steinbuch et al., 2018; Subedi et al., 2019a,b; Logviniuk and Fridman, 2020). A one-step synthesis of AK was developed to better compete with the commercially available crop fungicides and therapeutic antifungal agents (Figure 8; Subedi et al., 2019a). Among the compounds synthesized, the lead compounds **11a** and **11b** show strong antifungal activities, including activities against clinical strains of *C. auris* and *Aspergilla fumigatus* (Table 5). In pharmacokinetic studies in a mouse model, **11b** did not show signs of toxicity (e.g., body weight loss) up to the highest doses tested (52 mg/kg) (Subedi et al., 2020b). The cost of **11a** and **11b** production is lower than those of **K20**, making them more competitive than the widely used azole-based antifungal agents. As a group, the antifungal AKs represent a significant breakthrough for the classical AGs as they allow for the repurposing of large stockpiles of kanamycin sulfate, which is otherwise clinically obsolete as an antibacterial agent.

**TABLE 5:** Summarized MICs (μg/mL) of **11a** and **11b**^b^.

(A) MICs of 11a and 11b against fungi (Subedi et al., 2020b)															
Compound	A	B	C	D	E	F	G	H	I		J		K		L
**11a**	32	4	8	8	4	4	8	8	16		16		4		4
**11b**	16	4	8	8	4	4	8	8	8		16		0.5		0.5
Voriconazole	1	32	≥256	0.125	0.125	8	ND^a^	ND	ND		ND		ND		ND

**(B) MICs of 11a and 11b against clinical fungal isolates (Subedi et al., 2020b)**															
**Compound**	**A**	**B**	**C**	**D**	**E**	**F**	**G**	**H**	**I**	**J**	**K**	**L**	**M**	**N**	**O**

**11a**	8	8	8	4	4	8	8	16	2	4	8	2	4	4	4
**11b**	4	8	8	4	0.5	1	4	8	2	2	2	8	8	4	2
fluconazole	2	32	ND^a^	>64	>64	>64	ND	ND	ND	ND	ND	ND	ND	0.06	0.125

**(A)** A: *A. flavus*, B: *F. graminearium* B4-5A, C: *C. albicans* 64124, D: *C. albicans* MYA2876, E: *C. neoformans* H99, F: *R. pilimanae*; G: *C. albicans* B-311, H: *C. rugosa* 95-967, I: *C. parapsolis* Cas08-0490 (azole resistant), J: *C. tropicalis* 95-41, K: C. *auris* DI17-48, L: C. *auris* DI17-47.

^a^ND, not determined.

**(B)** A: *C. parapsilosis* (QC ATCC 22019), B: *C. krusei* (QC ATCC 6258), C: *Paecilomyces variotii* (QC), D: *C. albicans* (CA3), E: *C. auris* (DI17-47), F: *C. auris* (DI17-46), G: *A. fumigatus* (AF1), H: *A. fumigatus* (AF3), I: *Lomentospora prolificans* (LP1), J: *Scedosporium apiospermum* (SA1), K: *Scedosporium boydii* (SB1), L: *Apophysomyces* (APO1), M: *Apophysomyces* (APO2), N: *Blastomyces dermatitidis* (BD1), O: *B. dermatitidis* (BD3).

^a^ND, not determined.

^b^Conducted by The University of Texas Health Science Center at San Antonio, contracted through NIH’s Division of Microbiology and Infectious Diseases (DMID) of NIAID.

## Amphiphilic kanamycins as connexin hemichannel inhibitors

Large stockpiles of AGs, mainly neomycin sulfate and kanamycin sulfate are available worldwide, but their clinical use is limited due to the prevalence of bacterial resistance. Thus, efforts have been devoted to repurposing AGs for other applications. These include AGs as therapeutic options for spinal muscular atrophy (SMA) (Mattis et al., 2006, 2009) and disorders associated with hyperactivity of connexin hemichannels (HCs) (Fiori et al., 2017). Following these examples, AKs have also been investigated as connexin HC inhibitors with the goal of identifying leads that do not have antibacterial activity, show reduced cytotoxicity, and target HCs formed by specific connexin isoforms.

Connexins are transmembrane proteins that constitute the key building blocks of connexin HCs. Gap-junction channels (GJCs) formed by the head-to-head docking of two HCs, one from each of two adjacent cells, play diverse and pivotal roles in development, intercellular communication, and regulation (Meşe et al., 2007; Nielsen et al., 2012). Abnormal increases and decreases in the activity of HCs and GJCs have been linked to numerous genetic and acquired disorders such as deafness (Lee and White, 2009), heart arrhythmias and infarcts (Shintani-Ishida et al., 2007; Schulz et al., 2015; Leybaert et al., 2017), and cancer (Naus and Laird, 2010; Banerjee, 2016). Connexin HCs are mostly closed, but abnormally increased HC activity in inherited or acquired disorders results in cell damage. However, there are more than twenty different connexin isoforms, which have different functional properties and regulation, and are expressed differentially in various cells, tissues and organs (Beyer et al., 2009). Thus, desirable inhibitors should be selective not only toward connexin HCs but also the HCs of interest.

Among the connexin isoforms, connexin 43 (Cx43) and connexin 26 (Cx26) have attracted particular attention. Cx43 is expressed in many organs, including heart, brain, liver, kidney, skin, and myometrium. Mutations of Cx43 have been reported to associate with acute and chronic diseases occurred in these organs (Delmar and Makita, 2012; Prakoura et al., 2018; Slavi et al., 2018; Xu et al., 2019; Yang et al., 2019; Ma et al., 2020), whereas Cx26 mutations can lead to deafness and/or skin disorders (Bruzzone et al., 2003; Lee and White, 2009).

**FIGURE 9:**
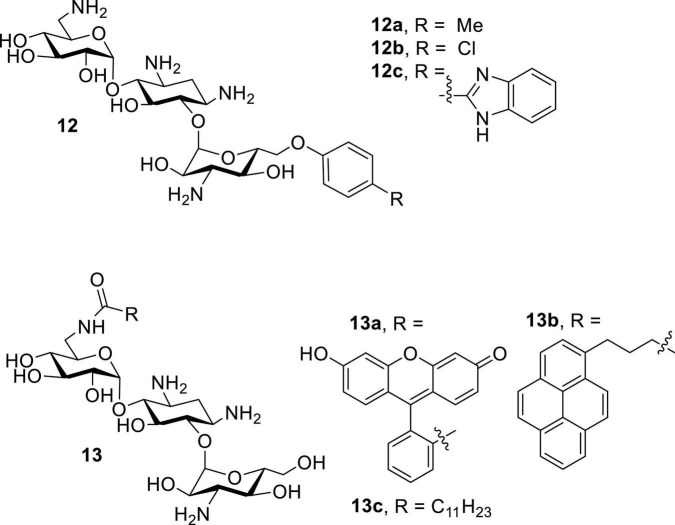
Amphiphilic kanamycin as connexin inhibitor.

Following the recent discovery that natural AGs inhibit connexin HCs without affecting GJCs (Figueroa et al., 2014), various classes of AKs were screened against Cx43 and Cx26 HCs (Alfindee et al., 2018; Subedi et al., 2020b). These AKs manifested inhibitory effects, and further SAR investigations revealed that several 6″- and 6′-modified AKs display inhibitory activities that can be tuned toward Cx43 HCs (Figure 9; Subedi et al., 2020a,^2021^). The results showed that compounds **12a** and **13c** were most selective toward Cx43 when compared to Cx26 HCs, with calculated selectivities (Cx26 IC_50_/Cx43 IC_50_) of ∼7 and ∼8, respectively (Table 6). These selectivities are ∼30-fold higher than that of kanamycin A (0.24). In addition, the AKs selected had no antibacterial activity and were not cytotoxic to HeLa cells at concentrations of up to 100 μM.

**TABLE 6:** IC_50_ of selected 6″ - and 6′-modified AKs toward Cx26 and Cx43 HCs.

AK class	Compound	Cx26 IC_50_ (μ M)	Cx43 IC_50_ (μ M)	Cx43/Cx26 Selectivity^a^
6″-modified	**12a**	49.4 ± 9.3	6.2 ± 1.4	7.97
	**12b**	17.2 ± 3.2	8.9 ± 1.6	1.93
	**12c**	6.0 ± 1.2	17.7 ± 5.6	0.34
6′-modified	**13a**	12.7 ± 2.2	8.9 ± 3.7	1.42
	**13b**	18.6 ± 2.1	8.6 ± 3.0	2.16
	**13c**	66.7 ± 6.9	9.7 ± 1.8	6.88
Kanamycin A		11.5 ± 1.8	48.0 ± 2.0	0.24

^a^The Cx43/Cx26 selectivity was calculated as IC_50_ Cx26/IC_50_ Cx43.

## Conclusions and future perspectives

In developed countries, AGs are often considered as a “last resort” for the treatment of serious bacterial infections. Semisynthetic approaches have been effective to modify naturally occurring AGs, yielding new AGs with revived antibacterial activity against AG-resistant bacteria. However, due to the rampage of bacteria equipped with diverse mechanisms of resistance such as AG-modifying enzymes, efflux pumps, decreases in membrane permeability and RNA target modifications, the semisynthetic approach still faces major challenges. The obvious problem is the cost of production. To combat the constantly evolving resistance mechanisms and complicated synthetic efforts, rare or expensive natural AGs have been employed as starting material. As a result, the end products are economically costly. Furthermore, these semisynthetic AGs still act by the same mechanisms as traditional AGs, binding to the 16S rRNA and interfering with protein synthesis, and thus, resistance evolves readily and soon after development of these new AGs. Finally, these AGs are likely to be less effective against bacteria such as *Enterococcus* or *Pseudomonas* sp., which are intrinsically resistant to AGs or equipped with flexible efflux resistance mechanisms, respectively.

AAGs offer an alternative solution to combat resistant pathogens. They can be prepared from cost effective AG feedstocks such as neomycin and kanamycin sulfates, which are mass produced by fermentation but have few clinical uses due to the resistance problem. Since AG-modifying enzymes are cellular enzymes, AAGs that target the bacterial membrane can be bactericidal without entering the cells, circumventing bacterial resistance conferred by AG-modifying enzymes. These two features, cost effective synthesis and bypassing a predominant bacterial mode of AG resistance, enable the potential uses of AAGs as effective antibacterials and for AG-based therapeutic uses previously limited by AG resistance. Additionally, AAGs exert no or much reduced cytotoxicity when compared to traditional AGs.

Highlights about AAGs in this review are that: (1) amphiphilic neomycins and neamine demonstrate potent antibacterial activities; (2) AKs have antibacterial as well as antifungal activities. The antifungal activity of AKs is of particular interest because traditional AGs are generally inactive against fungi and AKs employ novel membrane pore forming modes of action; (3) there are strong synergies between AKs and commercially used antifungal agents; (4) methods of cost-effective synthesis of lead AKs have been developed; and (5) AKs are connexin HC inhibitors. As such, AKs are potential therapeutics and experimental tools for deciphering the functions of HCs in human physiology and pathophysiology. AKs offer potential selective inhibition of HCs such as those formed by Cx43, and do not affect GJCs, thus paving the way for therapeutic use against disorders specifically due to hyperactive HCs.

Despite progress on the development and testing of AAGs, demonstration of efficacy *in vivo* is a crucial step toward the systemic applications of these compounds. To date, most of the reported AAGs have been studied only *in vitro* and a recent study showed that serum could prevent interactions between antimicrobial AAGs and plasma membranes (Logviniuk and Fridman, 2020). This report suggests limited use of AAGs as non-systemic drugs or for topical application. However, progress on formulations and carrier/delivery vehicles for AAGs or AAG modifications to reduce binding to serum proteins could improve clinical efficacy.

## Author contributions

All authors listed have made a substantial, direct, and intellectual contribution to the work, and approved it for publication.
